# FindPrimaryPairs: An efficient algorithm for predicting element-transferring reactant/product pairs in metabolic networks

**DOI:** 10.1371/journal.pone.0192891

**Published:** 2018-02-15

**Authors:** Jon Lund Steffensen, Keith Dufault-Thompson, Ying Zhang

**Affiliations:** Department of Cell and Molecular Biology, College of the Environment and Life Sciences, University of Rhode Island, Kingston, Rhode Island, United States of America; Universite Paris-Sud, FRANCE

## Abstract

The metabolism of individual organisms and biological communities can be viewed as a network of metabolites connected to each other through chemical reactions. In metabolic networks, chemical reactions transform reactants into products, thereby transferring elements between these metabolites. Knowledge of how elements are transferred through reactant/product pairs allows for the identification of primary compound connections through a metabolic network. However, such information is not readily available and is often challenging to obtain for large reaction databases or genome-scale metabolic models. In this study, a new algorithm was developed for automatically predicting the element-transferring reactant/product pairs using the limited information available in the standard representation of metabolic networks. The algorithm demonstrated high efficiency in analyzing large datasets and provided accurate predictions when benchmarked with manually curated data. Applying the algorithm to the visualization of metabolic networks highlighted pathways of primary reactant/product connections and provided an organized view of element-transferring biochemical transformations. The algorithm was implemented as a new function in the open source software package PSAMM in the release v0.30 (https://zhanglab.github.io/psamm/).

## Introduction

Metabolism forms the basis for understanding cellular processes in all living organisms. It comprises transformations of metabolites through biochemical reactions and can be viewed as a network graph, where metabolites are represented as individual vertices and reactions are represented as edges connecting the vertices. The reconstruction of metabolic networks can be applied to targeted pathways, species specific genome-scale models (GEMs) [1,2], or to represent an ensemble of metabolic potentials from all organisms [3]. In any case, metabolic reconstructions can quickly result in complex network topologies even for representing individual pathways in the central metabolism. This is due to the presence of multiple reactants and products in typical metabolic reactions, and the existence of hub metabolites (e.g. ATP/ADP, NAD/NADH, quinones, *etc*.) that are involved in a large number of metabolic processes. Deriving biological meaning from these networks, either through visual inspection or by analysis with algorithms, becomes difficult due to these complexities.

To facilitate the identification of biologically meaningful pathways, algorithms have been developed for reducing the complexity of metabolic network topology [4–9]. For example, the MetDraw algorithm [4] uses a heuristic approach where hub metabolites are identified as vertices having a vertex degree above a user-specified threshold. These hub metabolites usually represent common metabolites, such as energy currency compounds (e.g. ATP/ADP), cofactors, coenzymes, and small molecules (e.g. H_2_O), and they contribute to network complexity by creating links between different metabolic processes. In the MetDraw graph representation, the identified hub metabolites are shown as replicated vertices that associate with different reactions, hence eliminating the cross connections between different metabolic processes. This approach can be useful for providing an approximation of the traditional pathway diagrams, where the primary reactant/product pairs are used for tracing out individual metabolic processes. However, the MetDraw algorithm relies on the arbitrary determination of a degree threshold and is not feasible for visualizing the biosynthesis pathways of hub metabolites. A different approach is taken by other software that visualizes the reactant/product transformations as diagrams based on manual or semi-manual curations (e.g. Escher [10]; Arcadia [11]; Cytoscape [12]; CySBML [13]; ReconMap [14]; OptFlux visualization plugin [15]). These approaches are useful for making customized annotation of reactant/product pairs, but the requirement of extensive manual curations suggests that fully automated approaches are better suited for large-scale networks.

Examples of extensive manual curations of metabolic pathways are found in the KEGG [3] and the MetaCyc [16] databases, where the pathway diagrams present a simplified view of the pathways by leaving out some of the complexity of the network. The KEGG pathway maps are composed of static images manually constructed by expert curators according to the biochemical understandings of metabolite transformations. These diagrams often highlight the main chemical conversions that are relevant to the conventional understanding of biochemical pathways. However, they overlook the importance of additional metabolites (e.g. the above-mentioned currency compounds) in mediating the flux and directionality of metabolic reactions. In contrast, the pathway diagrams in MetaCyc provide a more detailed view of metabolic reactions that illustrate all participating metabolites. However, these pathway diagrams contain a focused view of individual processes and their global connections to the overall metabolism is frequently missing.

Identification of element-transferring reactant/product pairs for individual reactions remains as one of the fundamental challenges in detecting biologically meaningful network connections. A number of approaches have been developed for the mapping of reactant/product pairs based on the chemical structures of the metabolites [17–20]. The KEGG RPAIR database identifies element-transferring reactant/product pairs based on the automatic recognition of common metabolite structures and the expert-guided curation of chemical transformation patterns in individual reactions [18,21]. It provides extensive annotations of the reactions in the KEGG database and is by far one of the most extensive reference data set available. The MetaCyc database contains atom-mapping data for many reactions based on analyses of metabolite chemical structures [16,17]. This provides another extensive reference set of element-transferring reactant/product pairs. However, both the KEGG RPAIR and the MetaCyc atom-mapping annotations are restricted to metabolic reactions within their corresponding reaction databases. The application of these existing annotations to new metabolic reconstructions could be challenging as it requires the mapping of new reactions and metabolites, e.g. from expert curated GEMs or from other metabolic databases like ModelSEED [22], to the KEGG and MetaCyc databases, respectively. Such mappings are not always available and are often time consuming to construct.

So far only a limited number of studies have aimed at addressing the problem of mapping element-transferring reactant/product pairs given only chemical formula information for large reaction sets. The MapMaker algorithm proposed by Tervo and Reed [23] uses a *mixed integer linear programming* (MILP) approach to predict element transfers between reactants and products of individual metabolic reactions. Unlike the KEGG RPAIR and the MetaCyc atom-mapping annotations, MapMaker does not rely on information of metabolite structures but instead only requires the metabolite formulas. While the algorithm can potentially be applied for identifying reactant/product pairs that transfer any elements, the authors mainly focus on the application of the algorithm for predicting carbon-transferring pairs. The authors also created a manually curated set of carbon-transferring reactant/product pairs in the *Escherichia coli* GEM, iJO1366 [1]. This data set serves as an additional reference for evaluating new approaches, with a specific focus on predicting the carbon-transferring reactant/product connections. The MapMaker algorithm is applicable for analyzing any metabolic network with the simple inputs of reaction equations and metabolite formulas. However, it is time consuming to run for large-scale networks or reaction data sets, such as the reaction collection in the KEGG [3] and the MetaCyc [16] databases.

To solve the problems with existing approaches, this study presents a new algorithm, named *FindPrimaryPairs*, for predicting element-transferring reactant/product pairs with high efficiency and accuracy. This algorithm accounts for the identification of both carbon-transferring and non-carbon transferring metabolite connections, and it was validated with the KEGG RPAIR database [21], the MetaCyc atom-mapping database [16,17], and with manual annotations by Tervo and Reed [23]. For simplification, the term “*primary pairs”* was used in this study to indicate reactant/product pairs that carry elements from the reactant to the product. The algorithm was implemented as a new function in the open source software package PSAMM [24], which was applied to demonstrate primary reactant/product connections in the visualization of central metabolic processes.

## Materials and methods

### *FindPrimaryPairs*: An algorithm for the prediction of primary pairs

**Fig 1:**
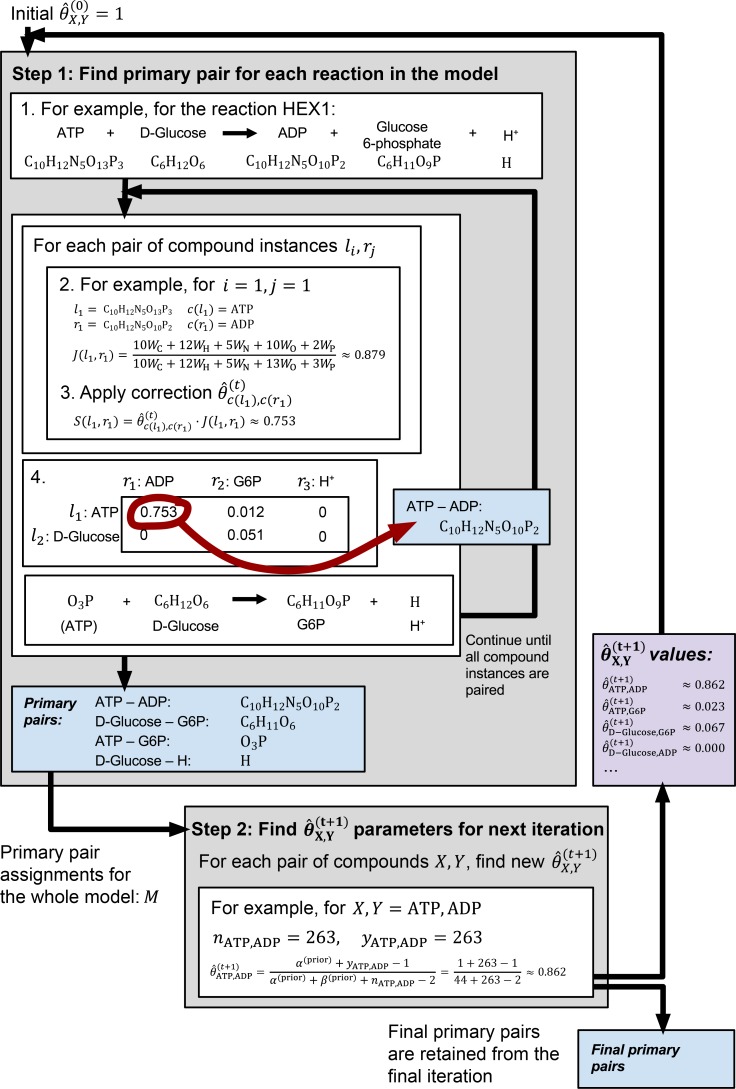
Diagram showing an application of the *FindPrimaryPairs* algorithm to a metabolic model.

The *FindPrimaryPairs* algorithm was designed to identify primary pairs from any given set of metabolic reactions (e.g. all reactions in a GEM or in any metabolic pathway database). It involves an iterative process of two major steps: (1) the identification of primary pairs for individual reactions based on a scoring function, and (2) the global refinement of a probability distribution estimate that contributes to the formulation of the scoring function. A detailed description of the *FindPrimaryPairs* algorithm is provided below and is illustrated with an example in Fig 1 (See the Results section for more details).

#### Step 1: Identify primary pairs for individual reactions

Given a reaction *R*_*i*_, a primary pair is a pair of interconverting compounds defined based on the procedure below:

1. Identify (*l*_1_, *l*_2_, ⋯, *l*_*s*_) and (*r*_1_, *r*_2_,⋯, *r*_*t*_) as lists of compound instances occurring respectively as reactants and products of reaction *R*_*i*_. These instances were expanded from sets of unique reactants and products based on the stoichiometric value. Let *c*(*x*) of instance *x* be the type of compound it was expanded from. The variables *s* and *t* indicate the number of compound instances on the two sides of the reaction equation.
2. For every compound pair, (*l*_*i*_, *r*_*j*_) with *i* ∈ (1, 2, ⋯, *s*) and *j* ∈ (1, 2, ⋯, *t*), calculate the weighted Jaccard similarity based on the compound formulas using Eq (1):
  $$J_{l_{i},r_{j}}=\frac{\sum_{e\in E}\left(\min\left(l_{i}^{\left(e\right)},r_{j}^{\left(e\right)}\right)∙W_{e}\right)}{\sum_{e\in E}\left(\max\left(l_{i}^{\left(e\right)},r_{j}^{\left(e\right)}\right)∙W_{e}\right)}\;fori\in \left(1,2,\ldots ,s\right)\;andj\in \left(1,2,\ldots ,t\right)\#$$ (1)
  where *E* is the set of all elements in *l*_*i*_ and *r*_*j*_, *x*^(*e*)^ is the count of element *e* in compound instance *x* (*x* being either *l*_*i*_ or *r*_*j*_), and *W*_*e*_ is a weight assigned to each element.
3. Perform a correction of the Jaccard similarity based on a value, ${\hat{\theta }}_{X,Y}$, which is a point estimate of the distribution *θ*_*X*,*Y*_ that models the probability of a compound pair (*X*, *Y*) being a primary pair in any reaction. The corrected score is defined in Eq (2), where *X* = *c*(*l*_*i*_) and *Y* = *c*(*r*_*j*_):
  $$S_{l_{i},r_{j}}=J_{l_{i},r_{j}}∙{\hat{\theta }}_{X,Y},\;for\;i\in (1,2,\cdots ,s)\;and\;j\in (1,2,\cdots ,t)\#$$ (2)
4. Pick a pair (l_*_, r_*_) with the highest $S_{l_{*},r_{*}}$ among all pairs, and assign (*X*, *Y*) as a primary pair where *X* = *c*(l_*_) and *Y* = *c*(r_*_). The transfer of elements between *X* and *Y* is defined as the count of all elements that are shared between l_*_ and r_*_.
5. Update the count of elements in formulas of l_*_ and r_*_ by removing the transferred elements as defined in step 4 from each formula. Repeat steps 2 to 5 with the updated formulas until the transfer of all elements between two sides of the reaction *R*_*i*_ has been accounted for in the list of assigned primary pairs.

The result of the above procedure is a list of predicted primary pairs for the reaction *R*_*i*_. In addition, each primary pair is associated with a formula indicating the predicted counts of elements transferred between the pair. The predicted primary pairs are used to obtain ${\hat{\theta }}_{X,Y}$, which is a point estimate of *θ*_*X*,*Y*_, as defined in the next section.

#### Step 2: Iterative refinement of ${\hat{\theta }}_{X,Y}$

Given two compounds, *X* and *Y*, from the two sides of a metabolic reaction, *R*_*i*_, define:

$$M_{X,Y}^{\left(R_{i}\right)}=\{\begin{array}{l} 1,\;\mathrm{if}\;X,Y\;\mathrm{is}\;a\;\mathrm{primary}\;\mathrm{pair}\;\mathrm{in}\;\mathrm{reaction}\;R_{i}\\ 0,\;\mathrm{otherwise} \end{array}\#$$ (3)

Therefore, $M_{X,Y}^{\left(R_{i}\right)}$ is an incidence of *M*_*X*,*Y*_ observed in the reaction, *R*_*i*_.

Next, define

$$\theta_{X,Y}=P_{\left(M_{X,Y}=1\right)}\#$$ (4)

Hence, *θ*_*X*,*Y*_ is the probability of *X* and *Y* being primary pairs in any reactions. A beta distribution is used to model *θ*_*X*,*Y*_:

$$\theta_{X,Y}∼Beta(\alpha_{X,Y},\beta_{X,Y})$$ (5)

where ${\hat{\theta }}_{X,Y}$ is estimated with the mode of the beta distribution:

$${\hat{\theta }}_{X,Y}=\frac{\alpha_{X,Y}-1}{\alpha_{X,Y}+\beta_{X,Y}-2}\#$$ (6)

Given a prior distribution, $\theta_{X,Y}^{\left(prior\right)}$, and a prediction of primary pairs, ${\overset{⃑}{m}}_{X,Y}$, in a set of reactions:

$$\theta_{X,Y}^{\left(prior\right)}∼Beta(\alpha^{\left(prior\right)},\beta^{\left(prior\right)})$$

$${\overset{⃑}{m}}_{X,Y}=(M_{X,Y}^{\left(R_{i}\right)}|i\in N_{X,Y}),M_{X,Y}^{\left(R_{i}\right)}\in \left\{0,1\right\}$$

In the above representation, *N*_*X*,*Y*_ is the set of reaction indices that reference reactions with compound *X* on one side and *Y* on the other. Let *n*_*X*,*Y*_ be the total number of reactions in *N*_*X*,*Y*_, and let *y*_*X*,*Y*_ be the number of instances where $M_{X,Y}^{\left(R_{i}\right)}=1,i\in N_{X,Y}$. The value of *y*_*X*,*Y*_ can be represented by Eq (7):

$$y_{X,Y}=\sum_{i\in N_{X,Y}}M_{X,Y}^{\left(R_{i}\right)}\#$$ (7)

The posterior distribution of *θ*_*X*,*Y*_ is estimated using the $\theta_{X,Y}^{\left(prior\right)}$ and ${\overset{⃑}{m}}_{X,Y}$ based on a binomial distribution model for *y*_*X*,*Y*_:

$$\theta_{X,Y}^{\left(posterior\right)}∼Beta(\alpha^{\left(prior\right)}+y_{X,Y},\beta^{\left(prior\right)}+n_{X,Y}-y_{X,Y})$$

Then, a point estimate ${\hat{\theta }}_{X,Y}$ is obtained using the mode of $\theta_{X,Y}^{\left(posterior\right)}$ following Eq (6), which corresponds to the *maximum a posteriori* (MAP) estimation.

The *FindPrimaryPairs* algorithm was applied to reaction sets by iteratively identifying primary pairs in individual reactions followed by a global refinement of the ${\hat{\theta }}_{X,Y}$ parameter according to primary pair predictions on all reactions (Fig 1). In each iteration, *t*, a MAP estimate of $\theta_{X,Y}^{\left(t\right)}$ was obtained as described above, an updated assignment of ${\overset{⃑}{m}}_{X,Y}^{\left(t\right)}$ was then identified using ${\hat{\theta }}_{X,Y}^{\left(t\right)}$ following the analysis of individual reactions, and a new estimate, ${\hat{\theta }}_{X,Y}^{\left(t+1\right)}$, was obtained based on ${\overset{⃑}{m}}_{X,Y}^{\left(t\right)}$ and $\theta_{X,Y}^{\left(t\right)}$. This iterative procedure continued until the point estimate, ${\hat{\theta }}_{X,Y}$, was stabilized for all compound pairs, as indicated in the following:

$$|{\hat{\theta }}_{X,Y}^{\left(t\right)}-{\hat{\theta }}_{X,Y}^{\left(t+1\right)}|<\epsilon \#$$ (8)

where ${\hat{\theta }}_{X,Y}^{\left(t\right)}$ and ${\hat{\theta }}_{X,Y}^{\left(t+1\right)}$ were point estimates of the posterior distributions from two successive iterations, and *ϵ* was a number close to zero (e.g. *ϵ* = 10^−5^). For the first iteration, the value of ${\hat{\theta }}_{X,Y}^{\left(0\right)}$ was set to 1. An example of applying the *FindPrimaryPairs* algorithm is illustrated in Fig 1 and described in the Results section.

### Parameter optimization

Optimized parameters of the *FindPrimaryPairs* procedure, including the weights of individual elements (*W*_*e*_) and the prior parameters of the beta distribution (*α*^(*prior*)^ and *β*^(*prior*)^), were identified based on analyzing the reactions in the KEGG database (Release 70.1). The RPAIR annotation for each reaction of the KEGG database was used to evaluate the prediction of both carbon-transferring and non-carbon transferring primary pairs. A confusion matrix was established for evaluating the predictions. The Matthews Correlation Coefficient (MCC) was used to measure the accuracy of predictions and was calculated as shown in Eq (9), where the number of true positive (TP), false positive (FP), true negative (TN), and false negative (FN) pairs were obtained from the confusion matrix.

$$MCC=\frac{TP∙TN-FP∙FN}{\sqrt{\left(TP+FP)(TP+FN)(TN+FP)(TN+FN\right)}}\#$$ (9)

The weights of individual elements (*W*_*e*_) were assigned with the consideration that carbon elements form the backbone of organic molecules and hence a similar number of carbon elements would indicate that two molecules are likely to be structurally similar. The hydrogen elements, in contrast, are peripheral to the molecule structure and are therefore less likely to predict structural similarity. Given this rationale, each carbon element was assigned a weight of 1, each hydrogen element was assigned a weight of 0, and all other elements (such as nitrogen, oxygen, phosphorus, etc.) were assigned a weight (*W*_*other*_) between these two extremes. A grid search was performed using the RPAIR annotations of the KEGG database as a reference dataset to identify the optimal values of parameters *α*^(*prior*)^, *β*^(*prior*)^, and *W*_*other*_. The range of *W*_*other*_ was assigned to decimal numbers between 0 and 1, with 51 steps of constant increments of 0.02, and the ranges of *α*^(*prior*)^ and *β*^(*prior*)^ were assigned to integers between 1 and 50. For each grid point, a confusion matrix was mapped based on the reference dataset and the parameters that resulted in the maximum MCC value were selected.

The primary pair prediction was applied to analyze metabolic reactions in the MetaCyc database and in a complete GEM, iJO1366, of the organism *E*. *coli*. The prediction was evaluated through the calculation of MCC values as defined in Eq (9), where the confusion matrix was constructed by comparing the primary pair predictions to the reference reactant/product pairs in the reference datasets [16,23]. Synthetic reactions like the biomass reactions were not considered in the evaluation because they represent artificial formulations of cellular processes. Since the iJO1366 reference data set included only pairs that transfer carbon, only primary pairs that were predicted to transfer at least one carbon element were compared to this dataset. In contrast, the comparison to the MetaCyc atom-mapping data considered all primary pairs.

### Software implementation

The *FindPrimaryPairs* algorithm was implemented as a function in the open source PSAMM software [24] and can be applied for analyzing GEMs or any given set of biochemical reactions. This function can be accessed with the “primarypairs” procedure of the “psamm-model” command by specifying the option “—method = fpp”. The new “primarypairs” procedure were made available from release v0.30 of PSAMM at https://zhanglab.github.io/psamm/.

As a comparison, an implementation of the *MapMaker* algorithm [23] was also included in the “primarypairs” procedure and can be accessed using the option “—method = mapmaker”. The *MapMaker* method relies on solving an MILP problem implemented on the linear programming solver framework of PSAMM. Multiple solvers, including the IBM ILOG CPLEX Optimizer, the Gurobi Optimizer, and the GNU Linear Programming Kit (GLPK), are compatible with this procedure. Specifically, in this study the *MapMaker* operations were performed using the CPLEX solver version 12.6.3.

### Visualization of a subnetwork of a genome-scale model

Metabolic networks analyzed in this study were visualized using a representation of bipartite graphs, where two sets of vertices were used to represent the compounds and reactions, respectively, and directed edges between the compound vertices and the reaction vertices were used to indicate the interconversion of compounds through reactions. Only carbon-containing compounds were included in the network visualization, and two different strategies were applied in the formulation of the network graphs. In a first strategy, each reaction vertex was represented only once in the graph and was associated with all carbon-containing reactants and products of the reaction. In a second strategy, reaction vertices were replicated to represent the connections within subsets of carbon-transferring reactant/product pairs identified based on the primary pair predictions. The visualization of pathway graphs was created in Cytoscape version 3.4.0 [12]. The compounds and reactions in conventional representation of the TCA cycle were laid out in a circular view and were presented with the same positioning of compound vertices. The remaining vertices in the graph were laid out using the spring-embedded approach in Cytoscape [12].

## Results

### Application of the *FindPrimaryPairs* algorithm

Fig 1 illustrates the application of the *FindPrimaryPairs* algorithm to the *E*. *coli* GEM, iJO1366 [1]. The algorithm follows an iterative process with the correction coefficient ${\hat{\theta }}_{X,Y}^{\left(t\right)}$ updated in every iteration until convergence (Materials and Methods). The two main steps of the iterative procedure are represented in the two gray boxes in Fig 1. The upper box demonstrates the identification of primary pairs from individual reactions, showing an example of the glucose kinase reaction, HEX1. The bottom box demonstrates the estimation of ${\hat{\theta }}_{X,Y}^{\left(t+1\right)}$ from ${\hat{\theta }}_{X,Y}^{\left(t\right)}$ and the formulation of a beta distribution, using an example of the reactant/product pair, ATP and ADP, and their primary pair assignments among all reactions of iJO1366.

The initial coefficient, ${\hat{\theta }}_{X,Y}^{\left(0\right)}$, was set to a fixed value of 1 in the implementation of the *FindPrimaryPairs* algorithm. This value was used for predicting the initial primary pair assignments, ${\overset{⃑}{m}}_{X,Y}^{\left(0\right)}$, in all reactions, and the ${\overset{⃑}{m}}_{X,Y}^{\left(0\right)}$ in turn determined the estimate, ${\hat{\theta }}_{X,Y}^{\left(1\right)}$, for the next iteration (Materials and Methods). Values in the upper gray box of Fig 1 show an example of the calculations made with ${\hat{\theta }}_{X,Y}^{\left(1\right)}$, for identifying primary pairs in the reaction, HEX1, and the lower gray box shows an example of obtaining an updated estimate, ${\hat{\theta }}_{ATP,ADP}^{\left(2\right)}$, based on the ATP–ADP pairing in all reactions of the iJO1366 model. Primary pairs in the reaction HEX1 were identified based on a five-step procedure described in Materials and Methods, which resulted in the assignment of four primary pairs that accounted for all element transfers from reactants to productions of the reaction (Fig 1, blue boxes). The same procedure was applied to each individual reaction in the iJO1366 model, and then the collection of all primary pairs from all reactions was used for determining ${\hat{\theta }}_{X,Y}^{\left(t+1\right)}$ for every compound pair that was present in the model (Fig 1, purple box). The iterative procedure continued until the value ${\hat{\theta }}_{X,Y}^{\left(t+1\right)}$ converged for every compound pair in the model (Materials and Methods).

### Grid search and selection of optimal parameters

**Fig 2:**
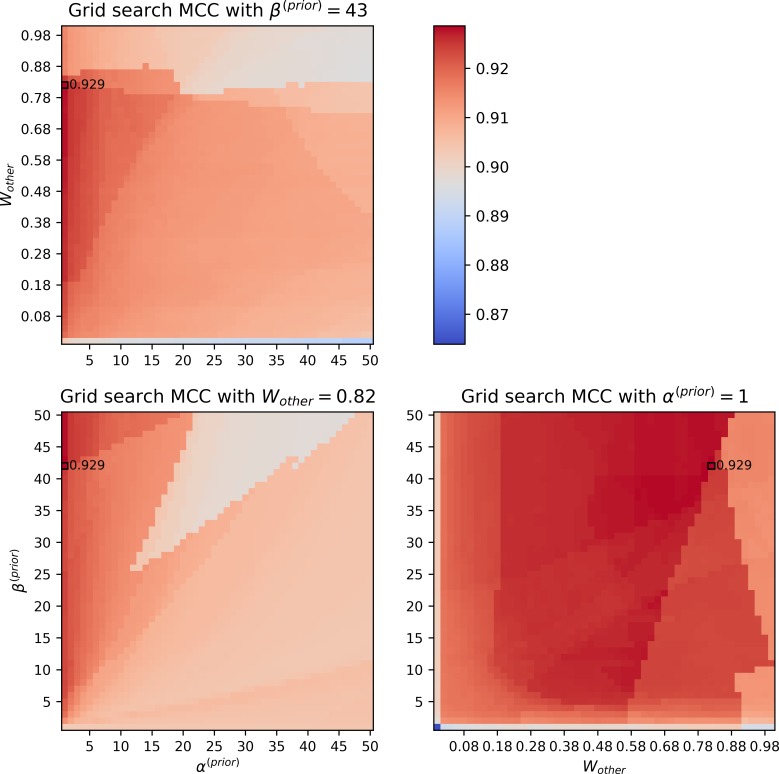
Heatmaps demonstrating pairwise relationships of the parameters, *W*_other_, *α*^(*prior*)^, and *β*^(*prior*)^, from a grid search of optimal values based on the RPAIR annotations of the KEGG database. The MCC values are shown with color coding at each step of the varying parameters (see the color legend on the upper right panel). The maximum MCC is marked with a black outline in each heatmap panel and labeled with its numeric value. In each heatmap, a selected parameter was fixed at its optimum (indicated in the title of each panel) and the remaining two parameters were varied within a given range of the grid search (x-axis and y-axis).

A grid search was performed for assigning the three parameters used in the *FindPrimaryPairs* procedure, including the weight of non-carbon, non-hydrogen elements (*W*_*other*_), and the values of prior parameters, *α*^(*prior*)^ and *β*^(*prior*)^ (Materials and Methods). Fig 2 provides snapshots of the grid search results, where each panel presents grids of two parameters at the optimal setup of a third parameter. The MCC values were used in grid search for identifying optimal parameters, and the KEGG RPAIR database [18,21] was used as a reference for evaluating the primary pair predictions (Materials and Methods).

The reference annotations used in the grid search analysis included 7569 biochemical reactions and 21174 reactant/product pairs that account for the chemical transformation of metabolites in the reactions. This reference dataset was created from the KEGG reaction database by eliminating unspecified reaction entries: First, 1380 reactions were skipped from the parsing of compound formulas because they involve compounds (549 out of 7827 compounds in the KEGG database) that could not be processed due to the lack of formula annotation, a variable number of elements in the formula, or a dot notation in the formula indicating complex chemical compositions. Second, 479 reactions were not considered because they have unbalanced non-hydrogen elements on the two sides of the reaction equations. Finally, 242 reactions were removed either because they are missing from the RPAIR annotation (55 reactions) or they involve at least one carbon-containing compound for which a pair is not identified in the RPAIR annotations (187 reactions).

According to the grid search, the MCC values were close to or higher than 0.9 within the tested range of all parameters (Materials and Methods). This indicated consistency of the *FindPrimaryPairs* procedure in predicting primary pairs even with varying parameter values. In other words, the choice of parameter values within the range had no significant influence on the final predictions. In searching for the optimal parameters, the MCC values appeared to decline slightly when the weight of non-carbon, non-hydrogen elements (*W*_other_) was above 0.88 or below 0.18, suggesting that an intermediate weight was favored for these elements. The optimal MCC was reached when *α*^(*prior*)^ approached 1. However, when both *α*^(*prior*)^ and *β*^(*prior*)^ were set to 1 so that the beta distribution model of *θ*_*X*,*Y*_ became a uniform distribution, the MCC value was lowest regardless of the setting of *W*_*other*_ (Fig 2, lower right panel). As a result, the grid search identified an optimal MCC value of 0.929 when the parameters *α*^(*prior*)^, *β*^(*prior*)^, and *W*_*other*_, respectively, approached 1, 43, and 0.82 (Table 1). These optimal parameters were applied in the implementation of the *FindPrimaryPairs* procedure and were used as the default parameters for our studies in the following sections.

**Table 1:** Parameter values applied in the default implementation of *FindPrimaryPairs*. The weight assignments of carbon (*W*_*C*_) and hydrogen (*W*_*H*_) elements were determined based on the design of the algorithm. The weight assignment of other elements *W*_*other*_, and the prior parameters *α*^(*prior*)^ and *β*^(*prior*)^ were determined based on a grid search of optimal parameters using the KEGG RPAIR annotations as reference data.

Parameter	Value
*W*_*C*_	1
*W*_*H*_	0
*W*_*other*_	0.82
*α*^(*prior*)^	1
*β*^(*prior*)^	43

### Comparing *FindPrimaryPairs* with *MapMaker* algorithms

The *FindPrimaryPairs* algorithm was further evaluated through a comparison with the *MapMaker* algorithm [23]. To achieve this, an implementation of both algorithms was constructed and applied to three reference datasets for evaluating the accuracy and efficiency of primary pair predictions (Materials and Methods). The first dataset included manually curated carbon-transferring reactant/product pairs of 2150 reactions in a complete GEM, iJO1366 [23]. The second dataset included both carbon-transferring and non-carbon transferring reactant/product pairs of the 7569 KEGG reactions annotated in the KEGG RPAIR database [18,21]. The third data set contained both carbon-transferring and non-carbon transferring reactant/product pairs of the 8452 MetaCyc reactions that had available atom mappings [16,17].

While both algorithms produced highly accurate predictions, *FindPrimaryPairs* achieved higher MCC values than the *MapMaker* algorithm when predicting primary pairs in all three reference datasets (Table 2). Of the 3688 carbon-transferring reactant/product pairs annotated in iJO1366, over 98% (3626 pairs) were successfully identified by the *FindPrimaryPairs* algorithm, while a slightly smaller fraction (3591 pairs; 97%) were correctly identified by the *MapMaker* algorithm. Of the 21174 reactant/product pairs in the KEGG RPAIR annotation, over 97% (20591 pairs) were correctly predicted by *FindPrimaryPairs*, while less than 95% (20113 pairs) were correctly predicted by *MapMaker*. Of the 23345 reactant/product pairs in the MetaCyc dataset, around 96% (22400 pairs) were correctly predicted by *FindPrimaryPairs*, while approximately 94% (21977 pairs) were correctly predicted by *MapMaker*. The numbers of false positive and false negative predictions generated by *FindPrimaryPairs* were also reduced as compared to the predictions generated by the *MapMaker* algorithm (Table 2). Hence, a combination of higher true positives and lower false predictions contributed to the improved accuracy of the *FindPrimaryPairs* algorithm.

**Table 2:** Comparing the accuracy and efficiency of *FindPrimaryPairs* and *MapMaker* algorithms using annotations in the iJO1366, the KEGG RPAIR database, and the MetaCyc atom-mapping data. The MCC values were calculated according to descriptions in Materials and Methods, and the running time (seconds) was calculated based on the average time cost in seven independent runs of each algorithm on each reference set. TP–true positive; FP–false positive; FN–false negative; TN–true negative.

Dataset	Method	TP	FP	FN	TN	MCC	Time (seconds)
**iJO1366**	***FindPrimaryPairs***	3626	60	62	1577	0.946	13.9
	***MapMaker***	3591	90	97	1547	0.918	70.9
**KEGG**	***FindPrimaryPairs***	20591	804	583	17214	0.929	52.4
	***MapMaker***	20113	1286	1061	16732	0.879	264.6
**MetaCyc**	***FindPrimaryPairs***	22400	1544	945	24248	0.899	76.1
	***MapMaker***	21977	1970	1368	23822	0.864	316.3

The *FindPrimaryPairs* approach also demonstrated significant improvement of running efficiency (Table 2), with an average running time of 13.9 seconds for processing the 2150 reactions in the iJO1366 GEM, 52.4 seconds for processing the 7569 reactions in the KEGG database, and 76.1 seconds for processing the 8452 reactions in the MetaCyc database. In contrast, it took the *MapMaker* approach at least four times longer to process the reactions in all three datasets.

### Using *FindPrimaryPairs* predictions for visualizing metabolic subnetworks

**Fig 3:**
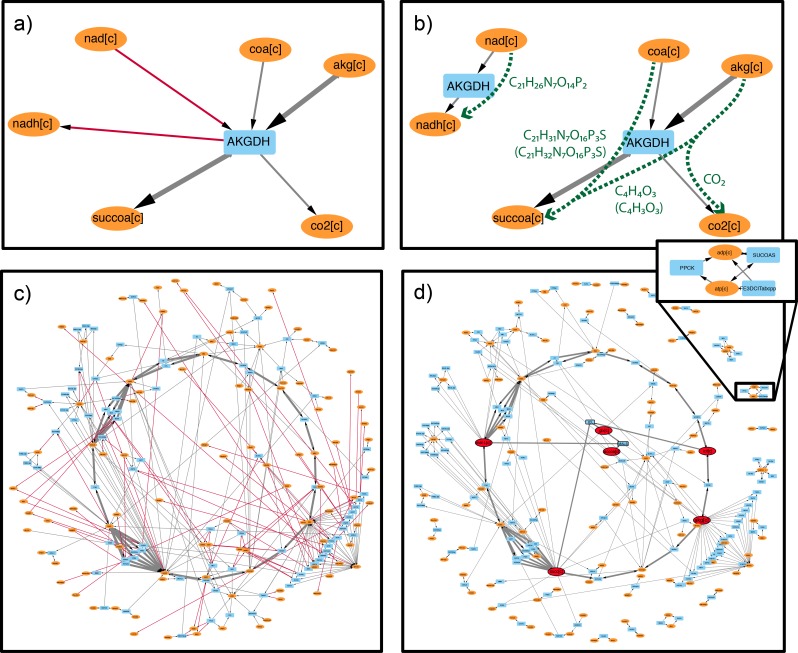
Bipartite graph representation of AKGDH (panels a and b) and a subnetwork (panels c and d) of the GEM, iJO1366. The panels a) and c) were represented using a conventional bipartite graph, where each reaction was shown as a single vertex connected with all compounds involved in the reaction. The panels b) and d) were similarly represented as bipartite graphs but converted reaction nodes to multiple vertices to highlight different primary pairs that carried out independent element transfers, e.g. as indicated with green dotted lines in panel b, where labels represent the predicted (no parentheses) or the annotated (in parentheses) element transfers. Unless specified, in all panels reaction nodes were shown as blue rectangles, compound nodes were shown as orange ovals, and the edge directions were assigned based on the annotation of reaction directions in the model. The edges with higher width indicated connections in the conventional representation of the TCA cycle, and the edges in red (panel c) represented independent primary pairs that would be isolated from the main element flow in the primary pair graph. The bordered compound nodes in red (panel d) highlighted network features visible in the primary pairs graph. Reaction abbreviations: AKGDH–2-Oxogluterate dehydrogenase; PPCK–Phosphoenolpyruvate carboxykinase; SUCOAS–Succinyl-CoA synthetase; FE3DCITabcpp–Iron transport from ferric-dicitrate via ABC system; ICL–Isocitrate lyase; MALS–Malate synthase. Compound abbreviations: akg–2-oxoglutarate; coa–coenzyme A; succoa–Succinyl-CoA; co2–Carbon dioxide; nad–Nicotinamide adenine dinucleotide; nadh–Reduced nicotinamide adenine dinucleotide; adp–Adenosine 5'-diphosphate; atp–Adenosine 5'-triphosphate; icit–Isocitrate; glx–Glyoxylate; mal-L–L-Malate; succ–Succinate; accoa–Acetyl-CoA. The suffix [c] indicated compounds located in the cytosol, and the suffix [p] indicated compounds located in the periplasm.

The *FindPrimaryPairs* algorithm was applied to reduce the complexity of network graphs. In Fig 3, a prediction of carbon-transferring compound pairs was applied for visualizing a subnetwork that represents the citric acid cycle (TCA cycle) and its metabolic contexts in a GEM, iJO1366 [1]. The subnetwork was constructed by first selecting nine main compounds that participate in the conventional representation of the TCA cycle (S1 Table) and then including additional reactions (S2 Table) and compounds (S1 Table) that are directly associated with these initial compounds. Visualization of the subnetwork was achieved with bipartite graph representations using two different strategies (Materials and Methods), which was demonstrated with an example of a single reaction (Fig 3A and 3B) as well as the entire subnetwork (Fig 3C and 3D).

A case study of the 2-Oxogluterate dehydrogenase reaction, AKGDH, was used to demonstrate differences in conventional bipartite graph representation (Fig 3A) as compared to the representation guided by primary pairs (Fig 3B). In the conventional bipartite graph, all participating compounds of a reaction were connected to the reaction vertex with directed edges. In contrast, subsets of compounds were identified in the primary pairs graph, and each subset was associated with an independent instance of the reaction vertex. In the AKGDH reaction, four primary pairs were identified using the *FindPrimaryPairs* algorithm (indicated with green dotted edges in Fig 3B), and each pair represented specific element transfers through the reaction (green labels in Fig 3B). The formulation of primary pair graph for AKGDH identified that the connection within one primary pair, NAD–NADH, was independent from other primary pairs, and hence it was associated with another instance of the reaction vertex. Therefore, the primary pairs graph effectively separated the representation of currency compounds (NAD and NADH) from the representation of element transfers among the substrates and products of a biochemical reaction.

The representation of a subnetwork associated with the TCA cycle was also constructed to demonstrate the application of primary pairs in visualizing complex connections from the central metabolism to other metabolic processes. In the conventional bipartite graph (Fig 3C), a large number of edges were directed across the center of the TCA cycle, reflecting the complex connections among different components of the subnetwork. This complexity was significantly reduced in the primary pairs graph (Fig 3D), where the connections between primary pathway compounds were isolated from the connections between currency compounds, such as ATP and ADP (Fig 3D, inset). Several additional features emerge from the primary pairs graph. For example, the compound 2-oxoglutarate (akg) was identified as an important hub to the downstream metabolic processes via connections to L-Glutamate (glu-L), which is a precursor of many biosynthesis pathways. The glyoxylate shunt was also more visible in the primary pair graph (Fig 3D). Hence, the visualization of primary pairs enhanced the biological interpretation of complex metabolic networks.

## Discussion

The complexity of metabolic networks prevents the identification of biologically meaningful features in the graph representation of metabolic transformations. Among various challenges the lack of accurate and efficient approach for detecting element-transferring reactant/product pairs is hindering the simplification of complex network topology. In this research, the new algorithm *FindPrimaryPairs* has been developed to perform automated prediction of primary pairs that carry out chemical transformation and element transfers in biochemical reactions. An implementation of the algorithm has been evaluated with the curated classifications of carbon-transferring pairs in a GEM, iJO1366 [1], and with the KEGG RPAIR and MetaCyc atom-mapping annotations that provide a global mapping of all element-transferring reactant/product pairs in biochemical reactions [16–18,21] Results from the evaluations have shown that *FindPrimaryPairs* achieved slightly better predictions than an existing algorithm, *MapMaker*, as indicated by their MCC values in mapping to reference datasets (Table 2). It is worth mentioning that the higher mapping accuracy is attributed not only to an increase in the number of true positive mappings but also to a reduction of false predictions by the *FindPrimaryPairs* algorithm. On all three reference datasets, the running time of *FindPrimaryPairs* has been reduced by at least four folds as compared to *MapMaker*. Additionally, the efficiency of the *FindPrimaryPairs* implementation could be further optimized by allowing parallel processing of independent reaction entries within each iteration of the global optimization (Fig 1).

While *FindPrimaryPairs* demonstrated enhanced accuracy and efficiency, it has some limitations similar to the *MapMaker* algorithm. First, both approaches rely on examining the similarity of metabolite formulas and would fail when reactant/product pairs of the highest formula similarity do not correspond to the biochemical mechanism of a reaction. For example, the Transaldolase reaction in iJO1366, TALA, transfers a dihydroxyacetone moiety from sedoheptulose 7-phosphate (s7p) to glyceraldehyde 3-phosphate (g3p), forming the products erythrose 4-phosphate (e4p) and fructose 6-phosphate (f6p). From the comparison of metabolite formulas, it appears that s7p should be paired with f6p and g3p be paired with e4p, but from analyzing the biochemical mechanism, a correct mapping of the reactant/product pairs should couple s7p with e4p, and g3p with f6p. Hence, the formula-based approach fails when a chemical transfer occurs between two substrates of similar element compositions, e.g. in the case of TALA, both substrates s7p and g3p are phosphorylated carbohydrates. However, these special case studies are not a major part of metabolic reaction databases. Since metabolite formulas are more readily available in metabolic databases than the interpretation of biochemical mechanisms, the formula-based approach represented by *FindPrimaryPairs* and *MapMaker* still provides significant advantage in analyzing large-scale metabolic networks.

Another problem that *FindPrimaryPairs* and *MapMaker* have in common comes from the possibility of having multiple optimal predictions of the primary pairs in a reaction. In the *MapMaker* algorithm, prediction of primary pairs is dependent on finding the solution of an MILP problem, which can result in multiple optimal solutions that lead to different pairings of the reactants and products. Only one solution can match the true mechanism of element transfer in a reaction, but the algorithmic design is not guaranteed to provide the correct solution in the presence of multiple solutions. Similarly, in the *FindPrimaryPairs* algorithm, multiple reactant/product pairs may have the same scores in comparing similarities of their formulas. Which pair is selected from the tie of scores may ultimately determine which one of the distinct predictions is reported. By default, ties of the highest similarity scores were broken in the PSAMM *FindPrimaryPairs* implementation by sorting the highest scoring pairs by metabolite names and selecting the first pair in the sorted list. This provides a way to consistently arrive at the same result when the *FindPrimaryPairs* algorithm is applied, but resolving the underlying issue of multiple equivalent solutions still requires manual curations.

**Fig 4:**
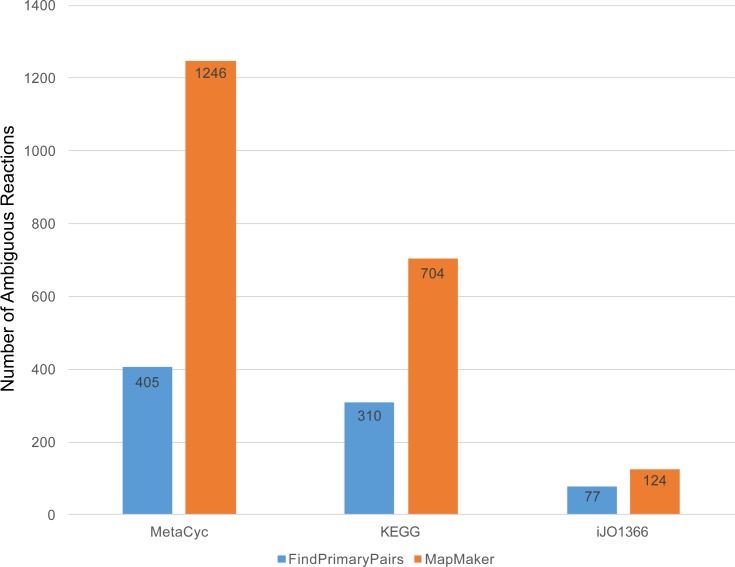
Bar chart showing the number of ambiguous reactions in the MetaCyc, KEGG, and iJO1366 reference datasets, where the two algorithms, *FindPrimaryPairs* and *MapMaker*, would potentially make arbitrary predictions of primary pairs. For *FindPrimaryPairs*, the reactions were counted for which the algorithm encountered ties on the top scoring reactant/product pairs in the last iteration of the primary pair assignment. For *MapMaker*, the reactions were counted for which the MILP solver would provide more than one optimal solutions that result in different primary pair predictions.

To evaluate the extent by which *FindPrimaryPairs* and *MapMaker* are influenced by these uncertainties in their algorithmic design, extensive sampling was performed on the implementation of both algorithms to count the number of reactions for which an arbitrary decision could have been made (Fig 4). The results revealed both algorithms produce ambiguous primary pair predictions for a small subset of reactions in all three reference databases, iJO1366 [1], KEGG [18,21] and MetaCyc [16,17], which contain 2150, 7569, and 8452 reactions, respectively. Compared to the *MapMaker* algorithm, *FindPrimaryPairs* demonstrated reduced level of ambiguity (Fig 4). This was largely due to the iterative refinement of reactant/product similarity scores based on the global assignment of primary pairs. It is worth mentioning that the measurements of uncertainty in Fig 4 reflected an upper bound of prediction ambiguity for the *FindPrimaryPairs* algorithm, because all reactions that had ties in top scores of metabolites similarly were counted as ambiguous, while not all ties would result in different prediction of primary pairs. For example, if a reaction has two reactants, A and B, and two products, C and D, a tie could occur in the top scores of two potential pairs: A–C and B–D. However, because these pairs do not represent a different mapping of reactants and products, the tie has no influence on the primary pair prediction. In contrast, in Fig 4 the number of ambiguous cases counted for the *MapMaker* algorithm reflected a true evaluation of reactions that had different primary pair predictions in the sampling. Hence, the *FindPrimaryPairs* algorithm provides a more stable approach that produces consistent predictions for a higher fraction of reactions in the reference databases than the *MapMaker* algorithm.

The identification of primary pairs is useful for visualizing metabolic subnetworks of complete GEMs (Fig 3). Compared to a conventional bipartite graph representation, the primary pairs graph has advantages in revealing primary substrate/product connections and identifying biologically meaningful network features. While previous studies rely on either the arbitrary identification of hub metabolites using compound vertices degrees [4] or the manual curation of individual metabolic pathways [10–15], The *FindPrimaryPairs* approach is both fully automated and avoids the drawback of making arbitrary decisions on the cutoff of vertices degrees in identifying hub metabolites. Further, it also permits the visualization of chemical transformations across different metabolic processes in the global metabolism. The primary pairs graph can be used on its own or combined with other graph layout algorithms, such as the grid layouts proposed by [25] and [26], to further reduce visual clutters in complex metabolic networks. Since the predictions provided by *FindPrimaryPairs* also include transfers of elements other than carbon, it can be applied to visually explore the flow of any other biologically important elements, such as nitrogen, phosphorus or sulfur, in the global metabolic processes.

## Supporting information

S1 Table List of compounds included in the graph representation of a subnetwork in iJO1366. The column "TCA_cycle" indicates the nine main compounds that participate in the conventional representation of the citric acid cycle (labeled with "Yes").
(PDF)

S2 Table List of reactions included in the graph representation of a subnetwork in iJO1366. (PDF)
