# Supplementary material for: FindPrimaryPairs: An efficient algorithm for predicting element-transferring reactant/product pairs in metabolic networks
Source: PLoS One. 2018 Feb 15;13(2):e0192891. doi: 10.1371/journal.pone.0192891 (PMC5814024; doi:10.1371/journal.pone.0192891)
Supplement: S1 Table — The column "TCA_cycle" indicates the nine main compounds that participate in the conventional representation of the citric acid cycle (labeled with "Yes"). (PDF) [file pone.0192891.s001.pdf]

Table S1. List of compounds included in the graph representation of a subnetwork in iJO1366. The column "TCA_cycle" indicates the nine main compounds that participate in the conventional representation of the citric acid cycle (labeled with "Yes").

Compound_ID	TCA_Cycle	Compound_Name	Chemical_Formula
25aics[c]		(S)-2-[5-Amino-1-(5-phospho-D-ribosyl)imidazole-4-carboxamido]succin	C13H15N4O12P
26dap-LL[c]		LL-2,6-Diaminoheptanedioate	C7H14N2O4
2dmmq8[c]		2-Demethylmenaquinone 8	C50H70O2
2dmmql8[c]		2-Demethylmenaquinol 8	C50H72O2
2mcit[c]		2-Methylcitrate	C7H7O7
2sephchc[c]		2-succinyl-5-enolpyruvyl-6-hydroxy-3-cyclohexene-1-carboxylate	C14H13O9
34hpp[c]		3-(4-Hydroxyphenyl)pyruvate	C9H7O4
3mob[c]		3-Methyl-2-oxobutanoate	C5H7O3
3mop[c]		(S)-3-Methyl-2-oxopentanoate	C6H9O3
3php[c]		3-Phosphohydroxypyruvate	C3H2O7P
4abut[c]		4-Aminobutanoate	C4H9NO2
4abutn[c]		4-Aminobutanal	C4H10NO
4mop[c]		4-Methyl-2-oxopentanoate	C6H9O3
aacald[c]		Aminoacetaldehyde	C2H6NO
ac[c]		Acetate	C2H3O2
accoa[c]		Acetyl-CoA	C23H34N7O17P3S
acg5sa[c]		N-Acetyl-L-glutamate 5-semialdehyde	C7H10NO4
acon-C[c]	Yes	cis-Aconitate	C6H3O6
acon-T[c]		trans-Aconitate	C6H3O6
acorn[c]		N2-Acetyl-L-ornithine	C7H14N2O3
adp[c]		ADP	C10H12N5O10P2
aicar[c]		5-Amino-1-(5-Phospho-D-ribosyl)imidazole-4-carboxamide	C9H13N4O8P
akg[c]	Yes	2-Oxoglutarate	C5H4O5
akg[p]		2-Oxoglutarate	C5H4O5
ala-L[c]		L-Alanine	C3H7NO2
amp[c]		AMP	C10H12N5O7P
arg-L[c]		L-Arginine	C6H15N4O2
argsuc[c]		N(omega)-(L-Arginino)succinate	C10H17N4O6
asp-L[c]		L-Aspartate	C4H6NO4
asp-L[p]		L-Aspartate	C4H6NO4
atp[c]		ATP	C10H12N5O13P3
cit[c]	Yes	Citrate	C6H5O7
cit[p]		Citrate	C6H5O7
co2[c]		CO2	CO2
coa[c]		Coenzyme A	C21H32N7O16P3S
cys-L[c]		L-Cysteine	C3H7NO2S
cyst-L[c]		L-Cystathionine	C7H14N2O4S
dcamp[c]		N6-(1,2-Dicarboxyethyl)-AMP	C14H14N5O11P
dhor-S[c]		(S)-Dihydroorotate	C5H5N2O4
dtdp4addg[c]		dTDP-4-amino-4,6-dideoxy-D-galactose	C16H26N3O14P2
dtdp4d6dg[c]		dTDP-4-dehydro-6-deoxy-D-glucose	C16H22N2O15P2
fe3dcit[p]		Fe(III)dicitrate	C12H10FeO14
fum[c]	Yes	Fumarate	C4H2O4
fum[p]		Fumarate	C4H2O4
gln-L[c]		L-Glutamine	C5H10N2O3
glu-L[c]		L-Glutamate	C5H8NO4
glx[c]		Glyoxylate	C2H1O3
hisp[c]		L-Histidinol phosphate	C6H11N3O4P
hkndd[c]		2-Hydroxy-6-oxonona-2,4-diene-1,9-dioate	C9H8O6
hkntd[c]		2-hydroxy-6-ketononatrienedioate	C9H6O6
hom-L[c]		L-Homoserine	C4H9NO3
iasp[c]		Iminoaspartate	C4H3NO4
ichor[c]		Isochorismate	C10H8O6
icit[c]	Yes	Isocitrate	C6H5O7
ile-L[c]		L-Isoleucine	C6H13NO2
imacp[c]		3-(Imidazol-4-yl)-2-oxopropyl phosphate	C6H7N2O5P
leu-L[c]		L-Leucine	C6H13NO2

mal-L[c]	Yes	L-Malate	C4H4O5
mal-L[p]		L-Malate	C4H4O5
micit[c]		methylisocitrate	C7H7O7
mmcoa-S[c]		(S)-Methylmalonyl-CoA	C25H35N7O19P3S
mql8[c]		Menaquinol 8	C51H74O2
mqn8[c]		Menaquinone 8	C51H72O2
nad[c]		Nicotinamide adenine dinucleotide	C21H26N7O14P2
nadh[c]		Nicotinamide adenine dinucleotide - reduced	C21H27N7O14P2
nadp[c]		Nicotinamide adenine dinucleotide phosphate	C21H25N7O17P3
nadph[c]		Nicotinamide adenine dinucleotide phosphate - reduced	C21H26N7O17P3
oaa[c]	Yes	Oxaloacetate	C4H2O5
ohpb[c]		2-Oxo-3-hydroxy-4-phosphobutanoate	C4H4O8P
op4en[c]		2-Oxopent-4-enoate	C5H5O3
orot[c]		Orotate	C5H3N2O4
oxadpcoa[c]		3-Oxoadipyl-CoA	C27H37N7O20P3S
pep[c]		Phosphoenolpyruvate	C3H2O6P
phe-L[c]		L-Phenylalanine	C9H11NO2
phpyr[c]		Phenylpyruvate	C9H7O3
phthr[c]		O-Phospho-4-hydroxy-L-threonine	C4H8NO7P
ppa[c]		Propionate (n-C3:0)	C3H5O2
ppcoa[c]		Propanoyl-CoA	C24H36N7O17P3S
ppp9[c]		Protoporphyrin	C34H32N4O4
pppg9[c]		Protoporphyrinogen IX	C34H38N4O4
pser-L[c]		O-Phospho-L-serine	C3H6NO6P
ptrc[c]		Putrescine	C4H14N2
pyr[c]		Pyruvate	C3H3O3
q8[c]		Ubiquinone-8	C49H74O4
q8h2[c]		Ubiquinol-8	C49H76O4
sl26da[c]		N-Succinyl-LL-2,6-diaminoheptanedioate	C11H16N2O7
sl2a6o[c]		N-Succinyl-2-L-amino-6-oxoheptanedioate	C11H12NO8
sucarg[c]		N2-Succinyl-L-arginine	C10H17N4O5
succ[c]	Yes	Succinate	C4H4O4
succ[p]		Succinate	C4H4O4
succoa[c]	Yes	Succinyl-CoA	C25H35N7O19P3S
sucglu[c]		N2-Succinyl-L-glutamate	C9H10NO7
sucgsa[c]		N2-Succinyl-L-glutamate 5-semialdehyde	C9H11NO6
suchms[c]		O-Succinyl-L-homoserine	C8H12NO6
sucorn[c]		N2-Succinyl-L-ornithine	C9H15N2O5
sucsal[c]		Succinic semialdehyde	C4H5O3
tartr-D[c]		D-tartrate	C4H4O6
tartr-D[p]		D-tartrate	C4H4O6
tartr-L[c]		L-tartrate	C4H4O6
tartr-L[p]		L-tartrate	C4H4O6
taur[c]		Taurine	C2H7NO3S
thdp[c]		2,3,4,5-Tetrahydrodipicolinate	C7H7NO4
tyr-L[c]		L-Tyrosine	C9H11NO3
udpLa4n[c]		uridine 5''-diphospho-{beta}-4-deoxy-4-amino-L-arabinose	C14H22N3O15P2
udpLa4o[c]		UDP-4-keto-pyranose	C14H18N2O16P2
val-L[c]		L-Valine	C5H11NO2
