# Supplementary material for: FindPrimaryPairs: An efficient algorithm for predicting element-transferring reactant/product pairs in metabolic networks
Source: PLoS One. 2018 Feb 15;13(2):e0192891. doi: 10.1371/journal.pone.0192891 (PMC5814024; doi:10.1371/journal.pone.0192891)
Supplement: S2 Table — (PDF) [file pone.0192891.s002.pdf]

Table S2. List of reactions included in the graph representation of a subnetwork in iJO1366

Reaction_ID	Reaction_Equation	Enzyme_Name
3OXCOAT	coa[c] + oxadpcoa[c] => accoa[c] + succoa[c]	3-oxoadipyl-CoA thiolase
ABTA	4abut[c] + akg[c] => glu-L[c] + sucsal[c]	4-aminobutyrate transaminase
ACONIs	acon-T[c] <=> acon-C[c]	aconitate isomerase (spontaneous)
ACONTa	cit[c] <=> acon-C[c] + h2o[c]	aconitase (half-reaction A, Citrate hydro-lyase)
ACONTb	acon-C[c] + h2o[c] <=> icit[c]	aconitase (half-reaction B, Isocitrate hydro-lyase)
ACOTA	acorn[c] + akg[c] <=> acg5sa[c] + glu-L[c]	acetylornithine transaminase
ADSL1r	dcamp[c] <=> amp[c] + fum[c]	adenylsuccinate lyase
ADSL2r	25aics[c] <=> aicar[c] + fum[c]	adenylosuccinate lyase
AKGDH	akg[c] + coa[c] + nad[c] => co2[c] + nadh[c] + succoa[c]	2-Oxogluterate dehydrogenase
AKGt2rpp	akg[p] + h[p] <=> akg[c] + h[c]	2-oxoglutarate reversible transport via symport (periplasm)
ALATA_L	akg[c] + ala-L[c] <=> glu-L[c] + pyr[c]	L-alanine transaminase
ARGSL	argsuc[c] <=> arg-L[c] + fum[c]	argininosuccinate lyase
ASPO5	asp-L[c] + fum[c] => h[c] + iasp[c] + succ[c]	L-aspartate oxidase
ASPT	asp-L[c] => fum[c] + nh4[c]	L-aspartase
ASPTA	akg[c] + asp-L[c] <=> glu-L[c] + oaa[c]	aspartate transaminase
AST	arg-L[c] + succoa[c] => coa[c] + h[c] + sucarg[c]	Arginine succinyltransferase
CITL	cit[c] => ac[c] + oaa[c]	Citrate lyase
CITt3pp	cit[c] + h[p] => h[c] + cit[p]	citrate transport out via proton antiport (periplasm)
CITt7pp	succ[c] + cit[p] => cit[c] + succ[p]	Citrate transport via succinate antiport (periplasm)
CS	accoa[c] + h2o[c] + oaa[c] => cit[c] + coa[c] + h[c]	citrate synthase
DHORDfum	dhor-S[c] + fum[c] => orot[c] + succ[c]	Fumarate dependent DHORD
DTARTD	tartr-D[c] => h2o[c] + oaa[c]	D(-)-tartrate dehydratase
FE3DCITabcpp	atp[c] + h2o[c] + fe3dcit[p] => adp[c] + (2) cit[c] + fe3[c] + h[c] + pi[c]	iron transport from ferric-dicitrate via ABC system (periplasm)
FRD2	fum[c] + mql8[c] => mqn8[c] + succ[c]	fumarate reductase
FRD3	2dmmql8[c] + fum[c] => 2dmmq8[c] + succ[c]	fumarate reductase
FUM	fum[c] + h2o[c] <=> mal-L[c]	fumarase
FUMt2_2pp	fum[p] + (2) h[p] => fum[c] + (2) h[c]	Fumarate transport via proton symport (2 H) (periplasm)
FUMt2_3pp	fum[p] + (3) h[p] => fum[c] + (3) h[c]	Fumarate transport via proton symport (3 H) (periplasm)
GLUDy	glu-L[c] + h2o[c] + nadp[c] <=> akg[c] + h[c] + nadph[c] + nh4[c]	glutamate dehydrogenase (NADP)
GLUSy	akg[c] + gln-L[c] + h[c] + nadph[c] => (2) glu-L[c] + nadp[c]	glutamate synthase (NADPH)
HKNDDH	h2o[c] + hkndd[c] => h[c] + op4en[c] + succ[c]	2-hydroxy-6-ketonona-2,4-dienedioic acid hydrolase
HKNTDH	h2o[c] + hkntd[c] => fum[c] + h[c] + op4en[c]	2-hydroxy-6-ketononotrienedioate hydrolase
HSST	hom-L[c] + succoa[c] => coa[c] + suchms[c]	homoserine O-succinyltransferase
HSTPT	glu-L[c] + imacp[c] => akg[c] + hisp[c]	histidinol-phosphate transaminase
ICDHyr	icit[c] + nadp[c] <=> akg[c] + co2[c] + nadph[c]	isocitrate dehydrogenase (NADP)
ICL	icit[c] => glx[c] + succ[c]	Isocitrate lyase
ILETA	akg[c] + ile-L[c] <=> 3mop[c] + glu-L[c]	isoleucine transaminase
LEUTAi	4mop[c] + glu-L[c] => akg[c] + leu-L[c]	leucine transaminase (irreversible)
MALS	accoa[c] + glx[c] + h2o[c] => coa[c] + h[c] + mal-L[c]	malate synthase
MALt2_2pp	(2) h[p] + mal-L[p] => (2) h[c] + mal-L[c]	Malate transport via proton symport (2 H) (periplasm)
MALt2_3pp	(3) h[p] + mal-L[p] => (3) h[c] + mal-L[c]	Malate transport via proton symport (3 H) (periplasm)
MALt3pp	mal-L[c] + h[p] => h[c] + mal-L[p]	L-malate transport out via proton antiport (periplasm)
MCITL2	micit[c] <=> pyr[c] + succ[c]	methylisocitrate lyase
MCITS	h2o[c] + oaa[c] + ppcoa[c] => 2mcit[c] + coa[c] + h[c]	2-methylcitrate synthase
MDH	mal-L[c] + nad[c] <=> h[c] + nadh[c] + oaa[c]	malate dehydrogenase
MDH2	mal-L[c] + q8[c] => oaa[c] + q8h2[c]	Malate dehydrogenase (ubiquinone 8 as acceptor)
MDH3	mal-L[c] + mqn8[c] => mql8[c] + oaa[c]	Malate dehydrogenase (menaquinone 8 as acceptor)
ME1	mal-L[c] + nad[c] => co2[c] + nadh[c] + pyr[c]	malic enzyme (NAD)
ME2	mal-L[c] + nadp[c] => co2[c] + nadph[c] + pyr[c]	malic enzyme (NADP)
MMM	succoa[c] => mmcoa-S[c]	Methylmalonyl-CoA mutase
MOX	mal-L[c] + o2[c] <=> h2o2[c] + oaa[c]	malate oxidase
OAADC	h[c] + oaa[c] => co2[c] + pyr[c]	oxaloacetate decarboxylase
OHPBAT	glu-L[c] + ohpb[c] <=> akg[c] + phthr[c]	O-Phospho-4-hydroxy-L-threonine:2-oxoglutarate aminotransferase
PHETA1	akg[c] + phe-L[c] <=> glu-L[c] + phpyr[c]	phenylalanine transaminase
PPC	co2[c] + h2o[c] + pep[c] => h[c] + oaa[c] + pi[c]	phosphoenolpyruvate carboxylase
PPCK	atp[c] + oaa[c] => adp[c] + co2[c] + pep[c]	phosphoenolpyruvate carboxykinase
PPCSCT	ppcoa[c] + succ[c] => ppa[c] + succoa[c]	Propanoyl-CoA: succinate CoA-transferase
PPPGO3	(3) fum[c] + pppg9[c] => ppp9[c] + (3) succ[c]	protoporphyrinogen oxidase (anaerobic)
PSERT	3php[c] + glu-L[c] => akg[c] + pser-L[c]	phosphoserine transaminase
PTRCTA	akg[c] + ptrc[c] => 4abutn[c] + glu-L[c]	Putrescine Transaminase
SDPDS	h2o[c] + sl26da[c] => 26dap-LL[c] + succ[c]	succinyl-diaminopimelate desuccinylase
SDPTA	akg[c] + sl26da[c] <=> glu-L[c] + sl2a6o[c]	succinyldiaminopimelate transaminase
SEPHCHCS	akg[c] + h[c] + ichor[c] => 2sephchc[c] + co2[c]	2-succinyl-5-enolpyruvyl-6-hydroxy-3-cyclohexene-1-carboxylate synthase
SGDS	h2o[c] + sucglu[c] => glu-L[c] + succ[c]	Succinylglutamate desuccinylase
SHSL1	cys-L[c] + suchms[c] => cyst-L[c] + h[c] + succ[c]	O-succinylhomoserine lyase (L-cysteine)
SOTA	akg[c] + sucorn[c] => glu-L[c] + sucgsa[c]	Succinylornithine transaminase
SSALx	h2o[c] + nad[c] + sucsal[c] => (2) h[c] + nadh[c] + succ[c]	succinate-semialdehyde dehydrogenase (NAD)
SSALy	h2o[c] + nadp[c] + sucsal[c] => (2) h[c] + nadph[c] + succ[c]	succinate-semialdehyde dehydrogenase (NADP)
SUCASPtpp	succ[c] + asp-L[p] <=> asp-L[c] + succ[p]	succinate:aspartate antiporter (periplasm)
SUCCt2_2pp	(2) h[p] + succ[p] => (2) h[c] + succ[c]	succinate transport via proton symport (2 H) (periplasm)
SUCCt2_3pp	(3) h[p] + succ[p] => (3) h[c] + succ[c]	Succintate transport via proton symport (3 H) (periplasm)

SUCCt3pp	succ[c] + h[p] => h[c] + succ[p]	succinate transport out via proton antiport (periplasm)
SUCDi	q8[c] + succ[c] => fum[c] + q8h2[c]	succinate dehydrogenase (irreversible)
SUCFUMtpp	succ[c] + fum[p] <=> fum[c] + succ[p]	succinate:fumarate antiporter (periplasm)
SUCMALtpp	succ[c] + mal-L[p] <=> mal-L[c] + succ[p]	succinate:malate antiporter (periplasm)
SUCOAS	atp[c] + coa[c] + succ[c] <=> adp[c] + pi[c] + succoa[c]	succinyl-CoA synthetase (ADP-forming)
SUCTARTtpp	succ[c] + tartr-D[p] <=> tartr-D[c] + succ[p]	succinate:D-tartrate antiporter (periplasm)
TARTD	tartr-L[c] => h2o[c] + oaa[c]	L(+)-tartrate dehydratase
TARTRt7pp	succ[c] + tartr-L[p] <=> tartr-L[c] + succ[p]	Tartrate/succinate antiporter (periplasm)
TAUDO	akg[c] + o2[c] + taur[c] => aacald[c] + co2[c] + h[c] + so3[c] + succ[c]	Taurine dioxygenase
TDPAGTA	dtdp4d6dg[c] + glu-L[c] => akg[c] + dtdp4addg[c]	dTDP-4-amino-4,6-dideoxy-D-glucose transaminase
THDPS	h2o[c] + succoa[c] + thdp[c] => coa[c] + sl2a6o[c]	tetrahydrodipicolinate succinylase
TYRTA	akg[c] + tyr-L[c] <=> 34hpp[c] + glu-L[c]	tyrosine transaminase
UDPKAAT	glu-L[c] + udpLa4o[c] <=> akg[c] + udpLa4n[c]	UDP-4''-ketopentose:UDP-4-amino-4-deoxy-L-arabinose aminotransferase
VALTA	akg[c] + val-L[c] <=> 3mob[c] + glu-L[c]	valine transaminase
